# Thanks to Reviewers 2020

**DOI:** 10.5334/pb.1055

**Published:** 2021-01-29

**Authors:** Editorial Team

**Affiliations:** 1 Psychologica Belgica, BE

Katarzyna Adamczyk

Marwa Nasser Alrajhi

Cristina Antunes

Elfi Baillien

Wivine Blekic

Cynthia Brown

Marc Brysbaert

Tinne Buelens

Jonathan Burnay

Gaëtane Caesens

Jeroen Camps

Séverine Casalis

Irene Cogliati Dezza

Desirée Colombo

Emiel Cracco

Egon Dejonckheere

Emeline Delaville

Ann DeSmet

Benoit Dompnier

Kate Ergo

Carole Fantini

Ghozlane Fleury

Sascha Fullbrunn

Sarah Galdiolo

Sandrine Gaymard

Sanne Ghielen

Donald Gibson

Philippe Golay

Lien Goossens

Tessa Haesevoets

Nurul Hartini

Rob Hartsuiker

Graham Hitch

Kathy Huet

Rotem Kahalon

Glenn Kiekens

Peter Kuppens

Christophe Leys

Baptist Liefooghe

Kate Mulgrew

Kristen Murray

Frédéric Nils

Guy Notelaers

Marco Ognibene

Santos Orejudo

Javier Ortuño Sierra

Karen Phalet

Etienne Quertemont

Isabel Raemdonck

François Ric

Arne Roets

Samuel Olayinka Salami

Hakan Sarıçam

Alexander Savi

Christina Schmidt

Céline Stassart

Marie Stievenart

Arnaud Szmalec

Sébastien Urben

Omer Van den Bergh

Nicolas Van der Linden

Anahi Van Hootegem

Stef Van Puyenbroeck

Tim Vantilborgh

Vasilis Vasiliou

Raquel Vazquez-Morejon

Aaron Veldre

Philippe Verduyn

Wouter Vleugels

